# Food substitutions revisited

**DOI:** 10.1093/ajcn/nqac222

**Published:** 2022-10-13

**Authors:** Daniel B Ibsen, Christina C Dahm

**Affiliations:** Medical Research Council Epidemiology Unit, Institute of Metabolic Science, University of Cambridge School of Clinical Medicine, Cambridge, United Kingdom; Steno Diabetes Center Aarhus, Aarhus, Denmark; Department of Public Health, Aarhus University, Aarhus, Denmark; Department of Nutrition, Sports and Exercise, University of Copenhagen, Frederiksberg, Denmark; Department of Public Health, Aarhus University, Aarhus, Denmark

See corresponding article on page 1379.

The concept of food substitutions is simple: eating 1 food instead of another. Whereas studying the effects of eating 1 food instead of another is typically explicit in interventional study designs, it is often implicit and sometimes hidden in analyses of observational studies. The substitution in question is often introduced as a consequence of adjusting regression models assessing associations between a food and an outcome of interest for total energy intake, thus restraining the comparison within the model to participants reporting the same energy intake. Willett et al. (1) discussed this in their seminal article, and the methods have been further discussed on theoretical and practical levels in several recent articles (2–4). Although intuition and practical subject matter knowledge are important, no formal simulation studies had been performed to date to investigate the implications of different food substitution modeling approaches. In this issue of *The American Journal of Clinical Nutrition*, Tomova et al. (5) fill this gap by investigating several modeling approaches for different food substitutions using simulations with parameters informed by the UK National Diet and Nutrition Survey. Of note, the authors investigate substitutions of 1 food instead of another as well as more complex substitutions of 1 food instead of several others. They also investigate the implications of modeling intake as nutrients, or as foods, either in calorie units or in mass units. Some of their findings confirm previous intuitions: we can rest assured. Others may require researchers to revisit how these models are applied in practice: a cause for concern.

## Rest assured

Food intake at a given time point can be conceptualized as a compositional exposure. Compositional data were defined by Arnold et al. (6) to “(…) comprise the parts of some whole, for which all parts sum to that whole.” Food substitution models take advantage of this feature to, more formally, estimate the relative causal effect of 1 food instead of another. In practice, there are several ways of specifying a food substitution, using either the leave-one-out method or the partition method (3). Tomova et al. in addition define an all-components method (4), which includes all the components that make up the whole. Although including all components is more straightforward when working with nutrients, because there tends to be fewer components, this can also be done with foods or food groups. In their study, simulations of single food substitutions showed that both the leave-one-out and the partition method could recover an estimate close to the true simulated relative causal effect, with 1 important caveat: given that the same unit was used throughout.

## Cause for concern

When using the leave-one-out method to specify the substitution of meat instead of fish, a commonly used model might include the investigated foods in grams and total energy in calories. The results from the simulation showed that mixing units of measurement reversed the sign of the regression coefficient, whereas models with the same units returned estimates close to the true simulated effect. Given that mixed-units models are often seen in nutritional epidemiology, these results should be a cause for concern. To examine this, we revisit a previous article in which we used such models to investigate substitutions of fish, poultry, or unprocessed red meat instead of processed red meat and risk of type 2 diabetes, adjusted for total energy intake (7). In our study, replacing 150 g/wk of processed red meat with fish, poultry, or unprocessed meat was associated with a slightly lower risk of type 2 diabetes using the leave-one-out method. Revisiting these analyses using the leave-one-out method, estimates were in different directions for mixed-unit (foods in grams and total energy in calories) and same-unit analyses (all in calories), but only for fish or poultry, not unprocessed red meat, instead of processed red meat (**Table 1**), thus confirming some, but not all, of our previous results. When we used the comprehensive all-components method, either per 150 g/wk or 100 kcal/d, we found similar estimates for each, and similar to the original mixed-unit analysis. Although we found similar associations in our reanalysis, it is important to note that the interpretation of the model with mixed units is, as Tomova et al. (5) call it, obscure.

**TABLE 1:** Food substitution models estimating the relative causal effect of fish, poultry, or unprocessed red meat instead of processed red meat in the Danish Diet, Cancer and Health cohort^1^

Estimand	Model		
	Name	Formula	Estimate, HR (95% CI)
Relative causal effect of fish instead of processed red meat	Mixed unit (150 g/wk), leave-one-out	T2D = *a*_0_ + *a*_1 URM_ + *a*_2 poultry_ + *a*_3 fish_ + *a*_4 total meat_ + *a*_5 TE_ + other^2^ foods + covariates^3^	
		R1.1 = *a*_3_	0.97 (0.94, 1.00)^4^
	Same unit (100 kcal/d), leave-one-out	T2D = *b*_0_ + *b*_1 URM_ + *b*_2 poultry_ + *b*_3 fish_ + *b*_4 total meat_ + *b*_5 TE_ + other foods + covariates	
		R1.2 = *b*_3_	1.02 (0.97, 1.08)
	Same unit (150 g/wk), comprehensive all-components	T2D = *c*_0_ + *c*_1 PRM_ + *c*_2 URM_ + *c*_3 poultry_ + *c*_4 fish_ + all other food groups^5^ + covariates	
		R1.3 = *c*_4_ − *c*_1_	0.97 (0.94, 1.00)
	Same unit (100 kcal/d), comprehensive all-components	T2D = *d*_0_ + *d*_1 PRM_ + *d*_2 URM_ + *d*_3 poultry_ + *d*_4 fish_ + all other food groups + covariates	
		R1.4 = *d*_4_ − *d*_1_	0.96 (0.90, 1.02)
Relative causal effect of poultry instead of processed red meat	Mixed unit (150 g/wk), leave-one-out	T2D = *e*_0_ + *e*_1 URM_ + *e*_2 poultry_ + *e*_3 fish_ + *e*_4 total meat_ + *e*_5 TE_ + other foods + covariates	
		R2.1 = *e*_2_	0.96 (0.93, 1.00)^4^
	Same unit (100 kcal/d), leave-one-out	T2D = *f*_0_ + *f*_1 URM_ + *f*_2 poultry_ + *f*_3 fish_ + *f*_4 total meat_ + *f*_5 TE_ + other foods + covariates	
		R2.2 = *f*_2_	1.03 (0.96, 1.10)
	Same unit (150 g/wk), comprehensive all-components	T2D = *g*_0_ + *g*_1 PRM_ + *g*_2 URM_ + *g*_3 poultry_ + *g*_4 fish_ + all other food groups + covariates	
		R2.3 = *g*_3_ − *g*_1_	0.97 (0.94, 1.00)
	Same unit (100 kcal/d), comprehensive all-components	T2D = *h*_0_ + *h*_1 PRM_ + *h*_2 URM_ + *h*_3 poultry_ + *h*_4 fish_ + all other food groups + covariates	
		R2.4 = *h*_3_ − *h*_1_	0.97 (0.90, 1.04)
Relative causal effect of unprocessed red meat instead of processed red meat	Mixed unit (150 g/wk), leave-one-out	T2D = *i*_0_ + *i*_1 URM_ + *i*_2 poultry_ + *i*_3 fish_ + *i*_4 total meat_ + *i*_5 TE_ + other foods + covariates	
		R3.1 = *i*_1_	0.97 (0.94, 1.00)^4^
	Same unit (100 kcal/d), leave-one-out	T2D = *j*_0_ + *j*_1 URM_ + *j*_2 poultry_ + *j*_3 fish_ + *j*_4 total meat_ + *j*_5 TE_ + other foods + covariates	
		R3.2 = *j*_1_	0.97 (0.92, 1.02)
	Same unit (150 g/wk), comprehensive all-components	T2D = *k*_0_ + *k*_1 PRM_ + *k*_2 URM_ + *k*_3 poultry_ + *k*_4 fish_ + all other food groups + covariates	
		R3.3 = *k*_2_ − *k*_1_	0.97 (0.94, 1.00)
	Same unit (100 kcal/d), comprehensive all-components	T2D = *l*_0_ + *l*_1 PRM_ + *l*_2 URM_ + *l*_3 poultry_ + *l*_4 fish_ + all other food groups + covariates	
		R3.4 = *l*_2_ − *l*_1_	0.97 (0.92, 1.02)

1 *n* = 53,135; incident cases, *n* = 6877; median follow-up time: 15.4 y. PRM, processed red meat; R, result; TE, total energy; T2D, type 2 diabetes; UPM, unprocessed red meat.

2 Other food groups: whole grains, fruits, vegetables, dairy products, potatoes, potato chips, soft drinks.

3 Covariates: BMI (in kg/m^2^; restricted cubic spline with 3 knots), waist circumference adjusted for BMI (continuous), smoking (never, former, current), alcohol (g/d; restricted cubic spline with 4 knots), education (<7 y, 8–10 y, >10 y), physical activity (≤ or >3.5 h/wk), sex (women or men, as strata), baseline date at study entry (tertiles, as strata), and age at baseline (tertiles, as strata).

4 Results from Ibsen et al. (7), although with slight differences in sample size and cases due to more variables being included.

5 All other food groups: other foods (legumes + refined cereals + eggs + nuts + vegetable oils + margarines + other animal fats + sweets + mayonnaise + soya + snacks), other drinks (fruit juices + vegetable juices + coffee + tea + water).

Another lesson from the simulation studies by Tomova et al. (5) is the complications that occur when specifying substitutions of 1 food with several others. This situation can be introduced “inadvertently” in models where total energy intake is adjusted for, but no substitution food or nutrient is specified. Again, reversal of coefficient signs occurred when mixing units. Furthermore, the all-components method could only estimate the average relative causal effect of meat (i.e., the effect of meat instead of a weighted average of all other foods) and none of the methods could sufficiently recover the relative causal effect of meat instead of cereal, dairy, fish, nuts, and other foods in grams.

## The continued evolution of food substitution methods

A key message from Tomova et al. (5) is that nutrition researchers ought to think carefully about the formulation of their research questions, the estimates of interest, and how the chosen statistical models reflect these. One way of doing this is to treat food intake as a compositional exposure and make this explicit in the chosen statistical model, which could be emphasized by using the all-components method. The article by Tomova et al. (5) encourages us to revisit our thinking of food substitution models, whether inadvertent or not. Future developments will want to focus on dietary changes and here the target trial framework (8) with g-methods (9, 10) to specify dietary interventions may aid in specifying food substitutions over multiple time points.

Nutrition epidemiology is often criticized for not providing valid results, but dietary guidelines and nutrition policies rely on such evidence for diseases of long latency and public health burden. We believe that a key reason for this criticism, which may stem from conflicting results from subsequent studies, is that the research questions and chosen methods in seemingly similar studies are often not precise enough to allow comparison. Given the complexity of diet as an exposure, there certainly is a need for further methodological developments to improve the methods to handle such complex questions. This article by Tomova et al. (5) is 1 such example.

## Data Availability

Data described in this article may be made available upon request pending on application to and approval by the Danish Cancer Society (e-mail: dchdata@cancer.dk)

## Notes

Author disclosures: The authors report no conflicts of interest

DBI is supported by the Independent Research Fund Denmark, grant 1057-00016B. CCD is supported by Aarhus University.

## References

[1] Willett WC , Howe GR, Kushi L. Adjustment for total energy intake in epidemiologic studies. Am J Clin Nutr. 1997;65(4):1220S–8S. 9094926 10.1093/ajcn/65.4.1220S

[2] Song M , Giovannucci E. Substitution analysis in nutritional epidemiology: proceed with caution. Eur J Epidemiol. 2018;33(2):137–40. 29478107 10.1007/s10654-018-0371-2

[3] Ibsen DB , Laursen ASD, Würtz AML, Dahm CC, Rimm EB, Parner ET et al. Food substitution models for nutritional epidemiology. Am J Clin Nutr. 2021;113(2):294–303. 33300036 10.1093/ajcn/nqaa315

[4] Tomova GD , Arnold KF, Gilthorpe MS, Tennant PWG. Adjustment for energy intake in nutritional research: a causal inference perspective. Am J Clin Nutr. 2022;115(1):189–98. 34313676 10.1093/ajcn/nqab266 PMC8755101

[5] Tomova GD , Gilthorpe MS, Tennant PWG. Theory and performance of substitution models for estimating relative causal effects in nutritional epidemiology. Am J Clin Nutr. 2022;116(5):1379–88. 10.1093/ajcn/nqac188 PMC9630885 36223891

[6] Arnold KF , Berrie L, Tennant PWG, Gilthorpe MS. A causal inference perspective on the analysis of compositional data. Int J Epidemiol. 2020;49(4):1307–13. 32154892 10.1093/ije/dyaa021 PMC7660155

[7] Ibsen DB , Warberg CK, Würtz AML, Overvad K, Dahm CC. Substitution of red meat with poultry or fish and risk of type 2 diabetes: a Danish cohort study. Eur J Nutr. 2019;58(7):2705–12. 30225630 10.1007/s00394-018-1820-0

[8] Hernan MA . Methods of public health research—strengthening causal inference from observational data. N Engl J Med. 2021;385(15):1345–8. 34596980 10.1056/NEJMp2113319

[9] Robins J . A new approach to causal inference in mortality studies with a sustained exposure period—application to control of the healthy worker survivor effect. Math Model. 1986;7(9–12):1393–512.

[10] Chiu Y , Chavarro JE, Dickerman BA, Manson JE, Mukamal KJ. Estimating the effect of nutritional interventions using observational data: the American Heart Association's 2020 Dietary Goals and mortality. Am J Clin Nutr. 2021;114(2):690–703. 34041538 10.1093/ajcn/nqab100 PMC8326054

